# Deletion of ARNT (Aryl Hydrocarbon Receptor Nuclear Translocator) in β-Cells Causes Islet Transplant Failure with Impaired β-Cell Function

**DOI:** 10.1371/journal.pone.0098435

**Published:** 2014-05-30

**Authors:** Amit Lalwani, Rebecca A. Stokes, Sue Mei Lau, Jenny E. Gunton

**Affiliations:** 1 Diabetes and Transcription Factors Group, Garvan Institute of Medical Research (GIMR), Sydney, Australia; 2 Faculty of Medicine, Westmead Hospital, University of Sydney, Sydney, Australia; 3 St Vincent’s Clinical School, University of New South Wales, Sydney, Australia; 4 Department of Diabetes and Endocrinology, Westmead Hospital, Sydney, Australia; Monash University, Australia

## Abstract

**Background:**

Replacing β-cells by islet-transplantation can cure type 1 diabetes, but up to 70% of β-cells die within 10 days of transplantation. ARNT (Aryl hydrocarbon Receptor Nuclear Translocator) regulates β-cell function, and potentially survival. Lack of ARNT impairs the ability of β-cells to respond to physiological stress and potentiates the onset of diabetes, but the exact role of ARNT in graft outcome is unknown.

**Aim:**

To investigate the effect of β-cell deletion of ARNT on graft outcomes.

**Methods:**

Islets were isolated from donor mice which had β-cell specific ARNT-deletion (β-ARNT) or littermate floxed controls. The islets were transplanted into diabetic SCID recipients in ratios of (a) 3 donors: 1 recipient, (b) 1 donor: 1 recipient or (c) ½ of the islets from 1 donor: 1 recipient. After 28 days, the kidney containing the graft was removed (nephrectomy) to exclude regeneration of the endogenous pancreas.

**Results:**

In the supra-physiological-mass model (3∶1), both groups achieved reasonable glycaemia, with slightly higher levels in β-ARNT-recipients. In adequate-mass model (1∶1), β-ARNT recipients had poor glucose control versus floxed-control recipients and versus the β-ARNT donors. In the low-β-cell-mass model (½:1) β-ARNT transplants completely failed, whereas controls had good outcomes. Unexpectedly, there was no difference in the graft insulin content or β-cell mass between groups indicating that the defect was not due to early altered β-cell survival.

**Conclusion:**

Outcomes for islet transplants lacking β-cell ARNT were poor, unless markedly supra-physiological masses of islets were transplanted. In the 1∶1 transplant model, there was no difference in β-cell volume. This is surprising because transplants of islets lacking one of the ARNT-partners HIF-1α have increased apoptosis and decreased islet volume. ARNT also partners HIF-2α and AhR (aryl hydrocarbon receptor) to form active transcriptional complexes, and further work to understand the roles of HIF-2α and AhR in transplant outcomes is needed.

## Introduction

Type 1 diabetes (T1D) is an autoimmune disease characterized by destruction of pancreatic β-cells and lack of endogenous insulin [1], [2]. Islet transplantation is a less invasive procedure than whole pancreas transplantation, and requires less immunosuppression [3], [4], [5]. However, at present, it still has lower long-term success rates than whole pancreas transplantation [5], [6], [7], [8], and insulin independence is achieved only when a sufficient number of islets are transplanted [1], [3].

The isolation and purification process inescapably induces devascularization and hypoxia in islets. Hypoxia is detrimental to β-cell function and survival and induces hypoxia-inducible factors (HIFs) [9], [10]. HIF-1 is a heterodimeric transcription factor composed of HIF-1α and Aryl hydrocarbon Receptor Nuclear Translocator (ARNT) [9], [11], [12]. We previously demonstrated that HIF-1α is important for β-cell survival and successful transplant outcomes [9]. In that setting, lack of HIF-1α leads to pronounced apoptosis of islets in the short-term after transplantation. ARNT is essential for the normal function of HIF-1α [13], [14], [15], [16].

ARNT (also called as HIF-1β) is expressed in islets, and is decreased in islets from humans with type 2 diabetes [11], [17], [18], [19]. It is important for normal angiogenesis, glycolysis and can be anti-apoptotic [13], [20], [21], [22]. ARNT also acts as the required dimerization partner for other members of the basic helix-loop-helix Per/AhR/ARNT/Sim (bHLH-PAS) family of transcription factors, including HIF-2α, HIF-3α and AhR [14], [23], [24], [25]. In this study the effect of deletion of ARNT in β-cells on islet transplantation outcomes was examined. Lack of β-cell ARNT causes very poor transplant outcomes, but unexpectedly, there is no significant decrease in either islet graft volume or insulin content at 28 days. This suggests that lack of β-cell ARNT does not increase immediate post-transplant apoptosis.

## Materials and Methods

### Ethics Approvals

All studies were approved by and conducted in accordance with the Garvan Animal Ethics Committee, application numbers #06.27 and #09.13. Minimum amounts of blood were collected via a distal tail nick for each required experiment.

### Animals

β-ARNT mice were created as previously reported [11], [26]. Transplant recipients were SCID mice (severe combined immunodeficiency) at 8–12 weeks of age. They were obtained from Animal Resources Centre (Canning Vale, WA). All animals were housed at the Garvan Biological Testing Facility, which employs a 12 hour on-off light cycle (0700–1900 on, 1900–0700 off). Mice were housed in standard filtered boxes with sterile bedding, standard chow food (Agrifood technology, Australia) (containing 59.9%, 26.7% and 13.4% calories from carbohydrate, protein and fat respectively) and water *ad libitum.*

### Diabetes Induction

SCID recipients were rendered diabetic with intraperitoneal streptozotocin (STZ; Sigma-Aldrich) in 10 mM citrate buffer, at a dose of 170 mg/kg. The blood glucose levels were determined using a glucometer (Freestyle Lite, Abbott, Australia). Mice that had blood glucose levels (BGL) of ≥20.0 mmol/L on ≥2 days were considered diabetic.

### Mouse Islet Isolation and Transplantation

**Figure 1:**
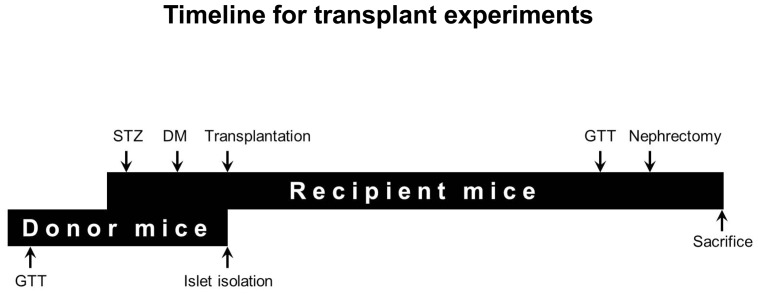
Timeline for transplant experiments. Donor mice undergo a GTT prior to islet isolation (∼3 days). Recipient mice are given an intraperitoneal injection of streptozotocin and diabetes occurrence is confirmed in approximately 5 days. Islets isolated from the donor mice are transplanted immediately into diabetic recipient mice. Recipient mice are checked for random-fed glucose levels at least 3 times a week until nephrectomy (28 days). GTT is performed on recipient mice on day 25 and nephrectomy on day 28 post-transplantation. Kidney with graft is collected for histology at nephrectomy.

The transplant model timeline is shown in **Figure 1**. Similar numbers of islets were isolated and purified from male donors as previously described [11]. In brief, donor mice were anaesthetised with an intraperitoneal injection of avertin. The pancreas was distended via the bile duct with 3 ml of 0.25 mg/ml Liberase-Enzyme Blend-RI (Roche, Indianapolis, IN, USA) in serum free islet isolation media, using a 30G needle. Each distended pancreas was digested at 37**°**C, after which M199 and 10% bovine calf serum (BCS) were added and tubes were alternately shaken and vortexed and then centrifuged to dislodge the acinar tissue from the islets. This process was repeated twice and the pellets were resuspended in 20 ml islet isolation media, and passed through a 425 micron sieve (US standard sieve, A.S.T.E. E-11 specifications dual MFG, Co. Chicago, IL, USA). The islets were separated on a Ficoll gradient (Ficoll-Plaque Plus 1.077 Amersham). Recipient mice received islets in (a) a supraphysiological ratio of islets from 3 donors to 1 recipient (3∶1), (b) a physiological ratio of islets from 1 donor to 1 recipient (1∶1) or (c) a minimal mass model of ½ the islets from 1 donor to 1 recipient (½:1).

Recipients were anaesthetised with 5% isoflurane in a chamber followed by maintenance of 1.5–2% isofluorane in oxygen. Anaesthesia depth was determined by footpad reflex testing. Islets were transplanted beneath the left kidney capsule and dispersed in a standardised manner. Graft function was determined by measuring random fed blood glucose levels (BGL) at least 3 times a week for 28 days. Pre transplant BGL are shown as day 0 for the recipients (p>0.3). Left nephrectomy was performed on day 28 post transplantation to confirm diabetes recurrence and to exclude regeneration of endogenous β-cells. Any mouse that was not diabetic by 2 days post nephrectomy (BGL ≥20.0 mmol/L) was excluded.

### Glucose Tolerance Test (GTT)

Glucose tolerance test (GTT) was performed on 1∶1 model donor mice at least 3 days prior to transplantation and on recipient SCID mice on day 25 post-transplantation as previously described (25,35). Mice were fasted overnight followed by an intraperitoneal injection of 20% dextrose at a dose of 2 g/kg. BGL was measured using the glucometer at 0, 15, 30, 60, 90 and 120 minutes post dextrose injection.

### Glucose Stimulated Insulin Secretion (GSIS)

The mice were fasted overnight. The following morning intraperitoneal glucose was given at 2 g/kg dose and the test was otherwise performed as previously reported (14,36). 15–20 µL of blood was collected at 0, 2, 5 and 20 minutes after the dextrose injection. Blood was immediately centrifuged and the supernatant (serum) was stored at −20°C for insulin ELISA assay.

### Mouse Islet Total Insulin Content and ELISA

Islets from the β-ARNT or control donor mice were isolated and lysed with 500 ul of RIPA buffer and samples were stored at −20°C. Insulin was measured by ELISA (Crystal Chem, Chicago, IL) as per manufacturer’s instructions.

### Immunohistochemistry (IHC)

Kidney grafts were collected from the recipient mice, and embedded in OCT (optimal cutting temperature) compound (Tissue-Tek, Sakura Finetek, CA). Sections were cut at 7 µm thickness, and blocked with 0.3% hydrogen peroxide.

To measure graft volume, every 6^th^ section was stained for insulin using rabbit anti-insulin antibody (Cell Signaling, Danvers, MA), anti-rabbit secondary antibody (DAKO, Carpinteria, CA), a peroxidase substrate containing 3,3-diaminobenzidine (DAB-brown) in a DAKO IHC autostainer. Sections were counterstained with hematoxylin on a Leica autostainer XL before mounting with MM 24 (Leica Biosystems, Nussloch, Germany) and a Leica auto-coverslip machine CV5030. Images were taken with a Leica DFC 450 camera on a Leica DM 4000 microscope.

### Immunofluorescence: Insulin and Caspase 3

Immunofluorescent staining of insulin and cleaved caspase 3 was performed as previously described [27]. Kidney grafts collected at day 28 post-transplantation (nephrectomy) were probed with primary antibodies; guinea pig polyclonal anti-insulin antibody diluted 1∶100 (Dako Cytomation, USA) and rabbit anti-cleaved caspase 3 antibody diluted 1∶100 (R&D systems, USA). Secondary antibodies included anti-guinea pig Alexa Fluor 488 (Invitrogen, Carlsbad, CA) and anti-rabbit Cy3 (DAKO, USA) both diluted at 1∶100 and DAPI diluted at 1∶1000 (DAKO, USA). Slides were observed under a Leica 5500 immunofluorescent microscope and images were taken with a Leica 6000 camera.

### Quantification of Cleaved Caspase 3 Staining

Individual caspase positive β-cells were counted in sections at day 28 post-transplantation. The data is represented as total number of caspase positive cells per insulin positive area of graft.

### Quantification of β-cell Mass

Pre-transplant β-cell mass for the mouse line is calculated as previously reported [11] by measuring β-cell area per area of whole sections of pancreas for at least 6 widely separated sections of pancreas per mouse. This percentage is then multiplied by pancreatic weight to obtain the β-cell mass in milligrams.

### Quantification of β-cell Volume

β-cell volume in the grafts was calculated using ImageJ 1.47 (NIH freeware) software. Freehand circling of the insulin positive β-cells was performed on every 6^th^ section of each kidney graft from first positive staining through to graft exhaustion. The β-cell volume was calculated using the trapezoidal rule, knowing the thickness of the sections.

### Quantification of Gene Expression by Real-time Quantitative PCR

RNA was isolated from grafts harvested at day 14 post-transplantation, using a Qiagen RNeasy Mini kit (Catalog number: 74106, Qiagen Valencia, CA) according to the manufacturer’s instructions. cDNA was generated from 0.5 µg of RNA and random hexamer primers using the Maxima First Strand cDNA Synthesis Kit for RT-qPCR (Thermo Scientific, MA, USA), as per manufacturer’s instructions and real time PCR was performed as previously reported [9], [28]. Gene expression of Bcl-2 associated X protein (*Bax*), B-cell lymphoma-extra large (*Bcl-xl*) (apoptotic pathway), glucose-6-phosphate isomerase (*G6pi*), glyceraldehyde 3-phosphate dehydrogenase (*Gapdh*), glucose transporter 2 (*Glut2*), *Aldolase*, glucokinase (*Gck*) (glycolytic pathway) and insulin receptor substrate 2 (*Irs2*), *Proinsulin* (insulin signaling) was measured using the Power SYBR green master mix (Applied Biosystems, Scoresby, Australia) and specific primers for each of the genes in an ABI Prism 7900HT Sequence Detection System (Applied Biosystems, Scoresby, Australia). TATA-box binding protein (*Tbp*) was used as an endogenous reference house-keeping gene. PCR primers are available on request. Following the reaction, fold change was calculated using the 2ΔΔCT method.

### Statistical Analysis

Data presented are mean ± SEM unless indicated otherwise. GraphPad Prism 6.0 (GraphPad Software, San Diego, CA) was used for data analysis and preparation of graphs. A *p*-value of <0.05 was considered statistically significant. Where multiple comparisons were made, Bonferroni post-hoc tests were used.

## Results

### Deletion of ARNT (Aryl Hydrocarbon Receptor Nuclear Translocator) in β-Cells Causes Islet Transplant Failure with Impaired β-cell Function

**Figure 2:**
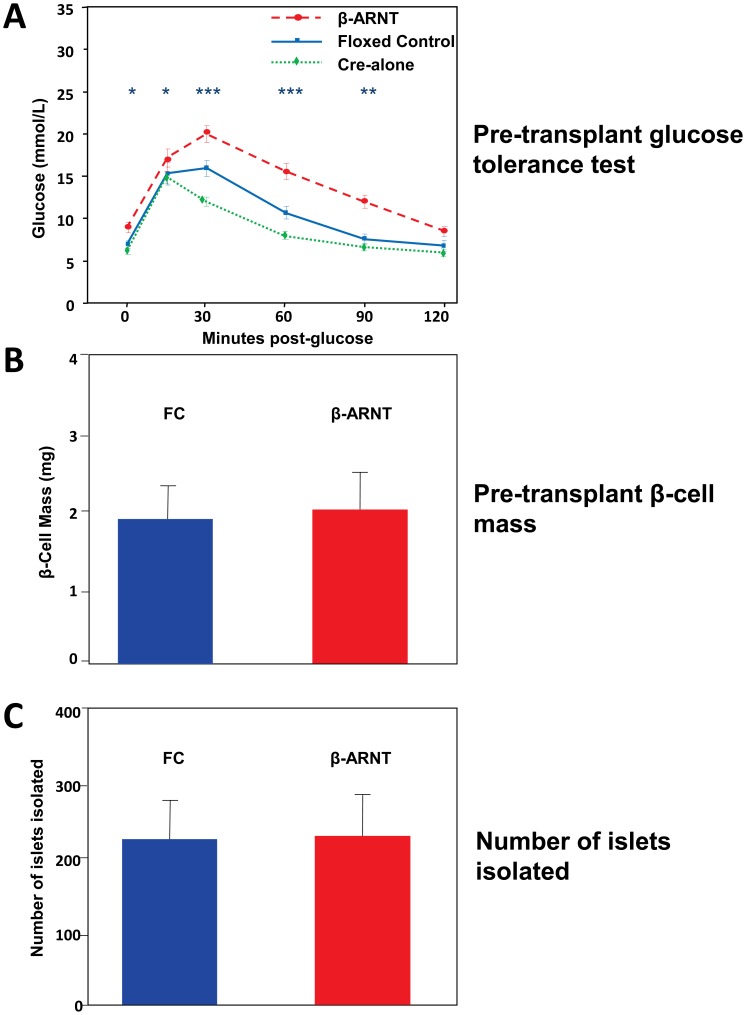
Pre-transplant glucose tolerance and β-cell mass and number of islets isolated. (**A**) β-ARNT donor mice had significant glucose intolerance compared to floxed controls and RIP-CRE alone mice (*** = p<0.001, ** = p<0.01, * = p<0.05). (**B**) Both β-ARNT and control mice show similar β-cell mass pre-transplantation. (**C**) Both β-ARNT and control mice had similar number of islets isolated. Average = 236 islets/β-ARNT mouse and 231 islets/control mouse. Data are presented as means ± SEMs.

Consistent with previous reports [11], [26], β-ARNT mice have mild glucose intolerance under normal conditions **(** **Figure 2A** **)**. The Cre-alone controls in this colony have slightly better glucose tolerance than the floxed controls (**Figure 2A**, green dotted line). Floxed control (FC) and β-ARNT donors have similar β-cell mass (**Figure 2B**) and similar numbers of islets were isolated from the mice (∼230 from each genotype) **(** **Figure 2C** **).**

### β-ARNT Islet Grafts Function Well when a Large Islet Mass is Transplanted

**Figure 3:**
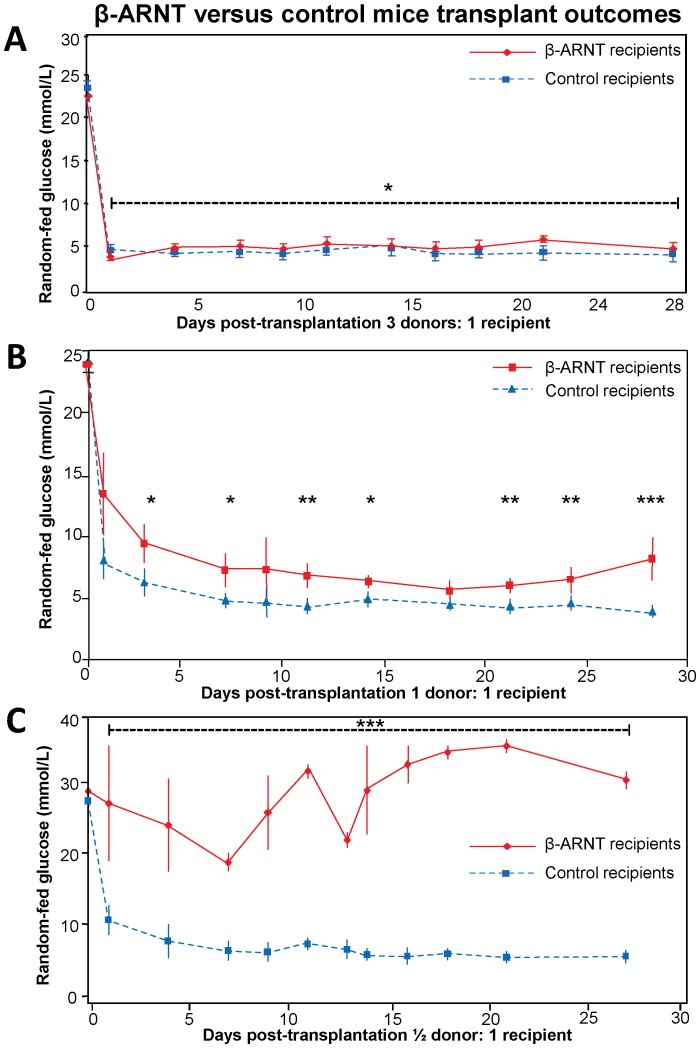
β-ARNT versus control mice transplant outcomes. (**A**) The 3∶1 transplant model shows that both β-ARNT and control recipients achieve reasonable glucose control suggesting that when a large mass of these islets are transplanted, even β-ARNT islets can function well (n = 4 per group) (* = p<0.05). (**B**) The 1∶1 transplant model shows that β-ARNT recipients have higher glucoses compared to control recipients (n = 6 per group) (*** = p<0.001, ** = p<0.01, * = p<0.05). (**C**) The ½:1 transplant model shows that β-ARNT recipients have severe glucose intolerance compared to control recipients (n = 4 per group) (*** = p<0.001). Data are presented as means ± SEMs.

Diabetic SCID mice were transplanted with islets from floxed control or β-ARNT mice in a 3 donors per recipient supra-physiological model. The β-ARNT recipients had lower BGL readings on day 1 after transplantation (3.8 versus 4.6 mmol/L, p<0.05) but subsequently showed mildly but significantly higher BGL readings (mean 4.9 versus 4.1 mmol/L, p = 0.02 ANOVA for repeated measures). These findings demonstrate that when a large mass of islets is transplanted, transplants of islets lacking β-ARNT function reasonably well (**Figure 3A**).

### Physiological Transplant Outcomes are Good for FC but not β-ARNT Recipients

To mimic the human transplant situation and allow comparison between donor and recipient glucose tolerance, a 1 donor: 1 recipient transplant model was tested. On average ∼230 islets were transplanted into each recipient. **Figure 3B** shows that random-fed blood glucose levels of floxed control recipients were normal (dashed-line), with a mean of 4.6 mmol/L over the study period. In contrast, β-ARNT recipients had worse glucose results, with mean levels being 63% higher (p = 0.0002). In β-ARNT recipients, BGLs also deteriorate towards the end of the 28 day period.

### β-ARNT Recipients had Complete Failure in ½:1 Transplant Model

A successful human islet isolation obtains ∼500,000 islet equivalents, from an estimated ∼1 million islets in a normal pancreas. A large proportion of these are thought to die in the post-transplant period [29], [30]. For this reason a ½:1 transplant model was tested. On average ∼115 islets (floxed control or β-ARNT) were transplanted into each diabetic recipient. With this decreased graft mass, FC recipients were still able to achieve and maintain completely normal BGL (dashed line, **Figure 3C**), demonstrating that a minimal mass of normal islets can cure diabetes. In marked contrast, recipients of the same mass of β-ARNT islets had complete failure with random fed BGL of >25.0 mmol/L (**Figure 3C**).

### β-cell ARNT is Required to Prevent Deterioration in Glucose Tolerance in Islet Recipients

**Figure 4:**
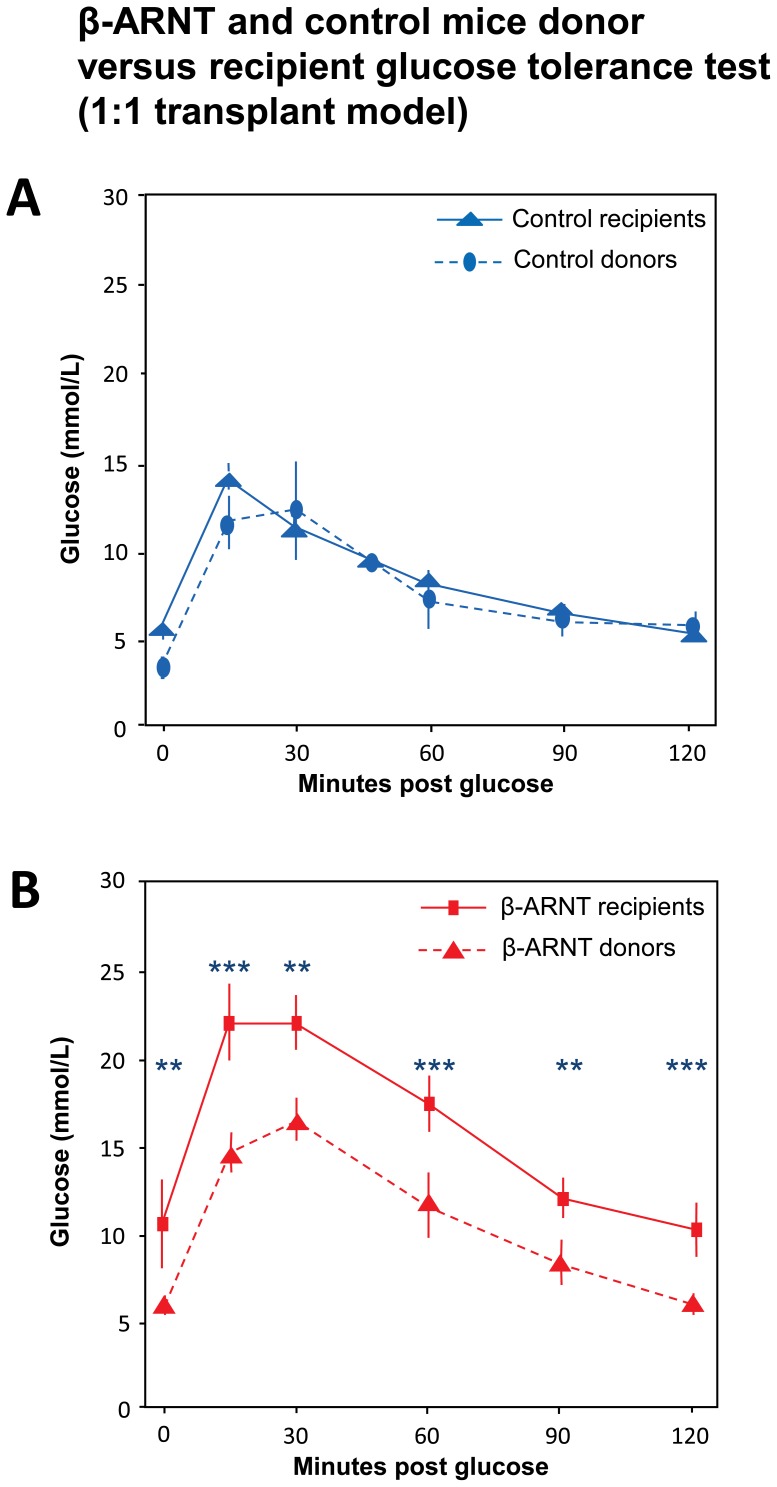
β-ARNT and control donor versus recipient mice glucose tolerance test (in 1∶1 transplant model). (**A**) Comparing glucose tolerance in both β-ARNT and control donors versus their recipients, the control recipients exhibit similar glucose tolerance compared to the control donors. (n = 4–6 per group) (**B**) Both β-ARNT recipients and donors have impaired glucose tolerance. (n = 4–6 per group) (*** = p<0.001, ** = p<0.01). Data are presented as means ± SEMs.

In the 1 donor: 1 recipient model, glucose tolerance in the donors was compared to glucose tolerance in the recipients of the islets. As shown in **Figure 4A**, control donors and their FC recipients have similar glucose tolerance test results. **Figure 4B** shows that, like in **Figure 2A**, β-ARNT donors have worse glucose tolerance than FC donors. However, unlike the FC mice, β-ARNT recipients have much worse glucose tolerance than their donors at all time points in the glucose tolerance test (**Figure 4B**).

### A Loss of ARNT was Found to be Associated with Glucose Intolerance with No Impact on Insulin Release in the β-ARNT Recipients

**Figure 5:**
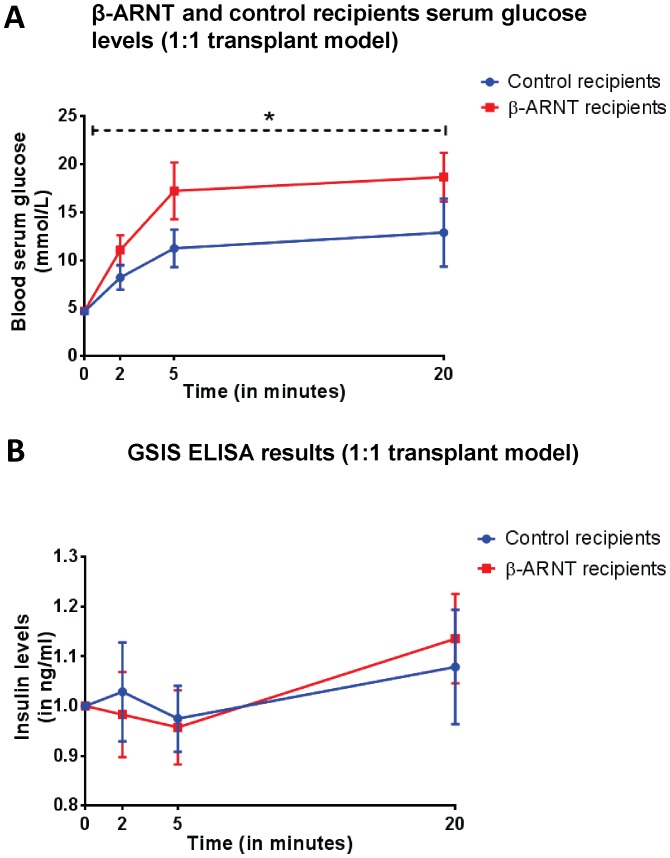
Glucose metabolism and insulin secretion in β-ARNT and control recipients at day 14 post-transplantation (in 1∶1 transplant model). (**A**) Serum glucose levels measured in the β-ARNT and control recipients tended to be higher in β-ARNT recipients on day 14 post transplantation. (n = 7–8 per group) (Two-way ANOVA test. *p* value = 0.0396). Data are presented as means ± SEMs. (**B**) Glucose stimulated insulin secretion measured (in ng/ml) by ELISA indicating no difference in the first and second phase of insulin secretion between the β-ARNT and control recipients at day 14. (n = 7–8 per group). Data are presented as means ± SEMs.

The 1∶1 transplant model as shown in **Figure 1** was repeated except nephrectomy was performed on day 14. β-ARNT recipients were slightly glucose intolerant compared to control recipients (**Figure 5A**). At 14 days post transplantation, there was no significant difference in the first phase and second phase insulin secretion between the control and β-ARNT recipients. However, these GSIS results are inappropriate for the much higher glucose results shown in **Figure 5A**, indicating that their insulin secretion is inappropriate for the glucose concentration.

### β-cells may not Necessarily Require ARNT for Normal Insulin Secretion and Adequate Expansion of β-cell Volume

**Figure 6:**
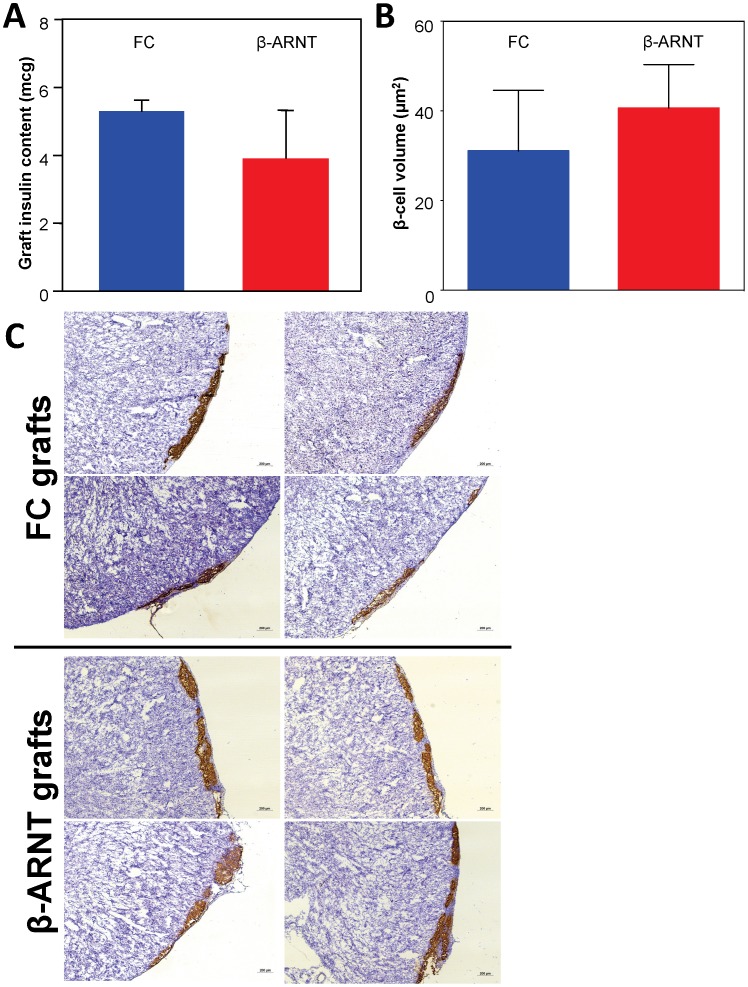
β-ARNT and control mice graft insulin content and β–cell volume (in 1∶1 transplant model). (**A**) There was no significant difference in total insulin content between β-ARNT and floxed control islets one week post-transplantation (n = 6 per group). (**B**) The β-cell volume was not significantly different between the control and β-ARNT transplants (n = 6 per group). Data are presented as means ± SEMs. (**C**) Representative photomicrographs of kidney graft sections from control recipients and β-ARNT recipients (n = 4 mice/group). Brown coloration indicates insulin producing β-cells and blue is the haematoxylin counter stain.

Previous studies showed that lack of one of ARNT’s partners, HIF-1α in β-cells causes impaired islet transplant outcomes with substantially decreased β-cell volume at 28 days. To determine whether lack of β-cell ARNT caused rapid graft loss, total insulin content of the kidney containing the graft was measured 1 week post-transplant. There was no difference in insulin content in the β-ARNT transplants compared to control transplants (**Figure 6A**).

Total β-cell volume was measured in the kidney grafts at day 28 (i.e. collected at nephrectomy). There was no significant difference in β-cell volume between control and β-ARNT transplants (**Figure 6B** **)**. Together, these two results unexpectedly suggest that unlike HIF-1α, ARNT does not play a role in short-term β-cell survival in islet transplantation. Interestingly, at 28 days, the grafts tended to have less intense insulin staining by immunohistochemistry in β-ARNT transplants compared to the floxed controls **(** **Figure 6C** **)**. This is consistent with the non-significantly lower total graft insulin content at 7 days, and can mark more rapid turn-over and release of insulin (decreased storage) when blood glucose levels are increased. Furthermore, apoptosis was assessed in the kidney grafts collected at day 28 post-transplantation (nephrectomy) by quantifying the number of caspase positive cells per mm^2^ of insulin graft area. There was no significant difference in the rate of apoptosis; FC mice had 0.1 caspase positive cells per mm^2^ of graft area; whereas the β-ARNT mice had no caspase positive cells in the slides examined.

### β-ARNT Recipients Show Altered Gene Expression in the Graft

**Figure 7:**
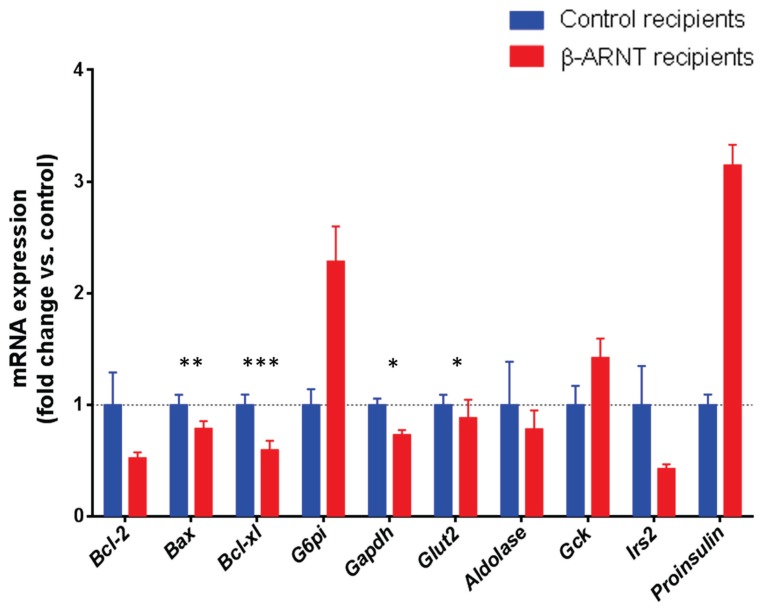
Gene expression changes in β-ARNT and control grafts. β-ARNT recipients (red bars) had a decrease in *Bcl-xl, Bax, Glut2* and *Gapdh* expression, a tendency to decreased *Irs2,* and to increased *Proinsulin* and *Gck* expression, as compared to the floxed control recipients (blue bars) (n = 7–8 per group). (*** = p<0.001, ** = p<0.01, * = p<0.05). Data are presented as means ± SEMs.

The graft was dissected from the day 14 nephrectomy samples for real-time PCR assessment of gene expression. Apoptotic and glycolytic pathways and insulin signaling were assessed. In the apoptotic pathway, there was a trend to decreased expression of the pro-apoptotic gene *Bax* **(** **Figure 7** **)**. The glucose transporter *Glut2* and the glycolytic gene *Gapdh* both showed significant decreases in β-ARNT grafts, consistent with impaired glucose stimulated insulin secretion (**Figure 7** **)**. Consistent with the suggestion of increased insulin in the graft (**Figure 5B** **and** **6C** **)**, the gene expression of pre-proinsulin was increased in the β-ARNT grafts. There was also decreased expression of the insulin signalling gene *Irs2*.

## Discussion

At present there are only two cures available for individuals with T1D; whole pancreas and islet transplantation. Islet transplantation is surgically less invasive and requires less intensive immunosuppression. There are ∼1 million islets in a normal healthy human pancreas [8], [31]. A good isolation may yield ∼500,000 islets. However, it is estimated that only 30–70% of transplanted islets may survive beyond 10 days post-transplantation [29], [30], [32]. Together, that would leave ∼15–20% of the original number of islets surviving in the recipient. Studies around the world have shown that ∼28–86% of normal glucose tolerant individuals who underwent a >50% pancreatectomy became glucose intolerant or diabetic [33], [34], [35]. This lack of absolute β-cell volume undeniably contributes to a gradual return to insulin therapy for many of the patients. β-cells are relatively susceptible to stress induced apoptosis, which contributes to their own demise [7], [31], [36]. Islets post-transplantation are exposed to various specific stresses including hyperglycaemia, IBMIR (instant blood mediated inflammatory reaction), cytokines and free radicals, lack of adequate nutrients, hypoxia and immune-mediated rejection (6). All of these factors are likely to be exacerbated in the long term, especially when insufficient β-cells are present at baseline.

Notably, despite the transfer of similar number of islets, β-ARNT recipients were unable to restore normoglycaemia and were highly glucose intolerant. The β-ARNT islets completely failed to control glucose in the ½:1 transplant model as compared to the 1∶1 or 3∶1 transplant model. This suggests that the absence of ARNT impairs the ability of β-cells to respond to physiological insults and contributes to the onset of diabetes and unsuccessful islet transplant outcome.

The lack of change in total insulin content and β-cell volume pre and post transplantation was surprising, given the decrease in graft volume with deletion of the ARNT-partner HIF-1α.

In both β-ARNT and control recipients, approximately half of the mice had impaired GSIS at 20 minutes, at this 14 day time. Delayed or impaired GSIS is common after transplantation. However, it is important to note that the control recipients had good glycaemic control, whereas the β-ARNT recipients did not, which means that their GSIS was inappropriate for their glucose. This suggests inadequate ability of the β-cells to sense glucose. Consistent with that, there were small, but statistically significant decreases in *Glut2* and *Gapdh* expression.

In contrast to our previous findings [9], with increased levels of apoptosis in β-HIF-1α null recipients, our current study shows that β-ARNT recipients did not have increased levels of apoptosis. ARNT acts as a general dimerization partner for class II members of the bHLH-PAS super family of transcription factors. Since ARNT is a partner for HIF-1α, in the absence of ARNT, HIF-1α should theoretically be unable to function normally. This suggests either a compensatory change with deletion of another ARNT-partner containing transcription factor, or that HIF-1α is binding to another transcriptional partner. If correct, what this potential partner could be remains unknown.

In conclusion, although much remains to be learned about β-cell biology and T1D, our findings show that ARNT plays an important role in graft function, but not graft volume post transplantation. The findings provide an interesting new insight into different roles for HIF-1α and ARNT in post-transplant β-cell survival. ARNT may be a therapeutic target for graft function. Identifying ways to increase ARNT in the pre- or post-transplantation period may improve islet function and therefore successful islet transplantation.
